# An optimized dynamic attribute-based searchable encryption scheme

**DOI:** 10.1371/journal.pone.0268803

**Published:** 2024-10-29

**Authors:** Shahzad Khan, Shawal Khan, Abdul Waheed, Gulzar Mehmood, Mahdi Zareei, Faisal Alanazi

**Affiliations:** 1 Department of Information Security, Military College of Signals (MCS), NUST, Islamabad, Pakistan; 2 Department of Computer Science, Shaheed Benazir Bhutto University Sheringal, Sheringal, Dir(U), Pakistan; 3 Department of Computer Science, COMSATS University Islamabad, Islamabad, Pakistan; 4 Department of Computer Science, Women University, Swabi, Swabi, Pakistan; 5 School of Electrical and Computer Engineering, Seoul National University, Seoul, South Korea; 6 Department of Computer Science, IQRA National University, Swat Campus, Odigram, Pakistan; 7 Tecnologico de Monterrey, School of Engineering and Sciences, Zapopan, Mexico; 8 Dept Elect Engn, Coll Engn, Prince Sattam Bin Abdulaziz Univ, Al Kharj, Saudi Arabia; University College of Engineering Tindivanam, INDIA

## Abstract

Cloud computing liberates enterprises and organizations from expensive data centers and complex IT infrastructures by offering the on-demand availability of vast storage and computing power over the internet. Among the many service models in practice, the public cloud for its operation cost saving, flexibility, and better customer support popularity in individuals and organizations. Nonetheless, this shift in the trusted domain from the concerned users to the third-party service providers pops up many privacy and security concerns. These concerns hindrance the wide adaptation for many of its potential applications. Furthermore, classical encryption techniques render the encrypted data useless for many of its valuable operations. The combined concept of attribute-based encryption (ABE) and searchable encryption (SE), commonly known as attribute-based keyword searching (ABKS), emerges as a promising technology for these concerns. However, most of the contemporary ABE-based keyword searching schemes incorporate costly pairing and computationally heavy secret sharing mechanisms for its realization. Our proposed scheme avoids the expensive bilinear pairing operation during the searching operation and costly Lagrange interpolation for secret reconstruction. Besides, our proposed scheme enables the updation of access control policy without entirely re-encrypting the ciphertext. The security of our scheme in the selective-set model is proved under the Decisional Bilinear Diffie-Hellmen (DBDH) assumption and collision-free. Finally, the experimental results and performance evaluation demonstrate its communication and overall efficiency.

## Introduction

In today’s modern age, cloud computing presents an appealing computing infrastructure that provides ubiquitous access using the internet. Nowadays’ cloud computing architecture comes in three architecture models: public, private, and hybrid models. However, many individuals are inclined towards the public cloud as they offer easy data sharing, personalized files, finance-related information, and healthcare information. Nevertheless, the companies that provide cloud services and consumers may not belong to the same trusted domain. As a result, the privacy and confidentiality of stored data on public cloud servers become a critical problem. Generally speaking, the data can be encrypted before outsourcing to protect the confidentiality and privacy of outsourced data. As the outsourcing of encrypted data creates significant challenges [1], other data users can not directly perform computation, searching operations, the users can not get the expected data by using the keyword searching method. Furthermore, data management and access control also become critical issues [2].

**Fig 1:**
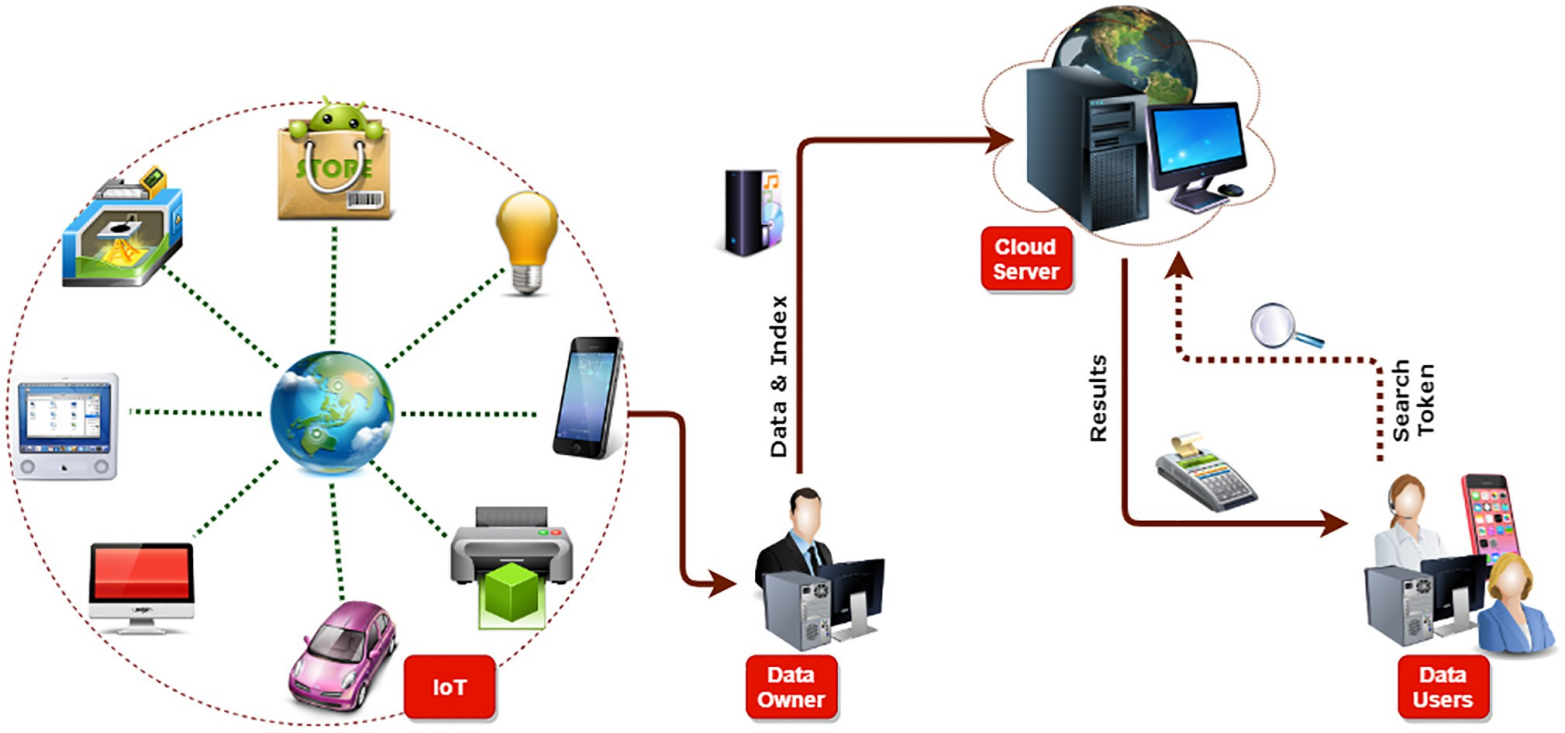
Searchable encryption.

Searchable Encryption (SE) has been widely adopted to overcome the problems mentioned earlier. The search operation is performed over ciphertext data without exposing the security and privacy of original data. The working mechanism of SE is depicted in Fig 1 where, first, the data owner outsources their ciphertext data to the cloud server to perform the search operation over encrypted data. The data users can send a search token to the cloud server, the cloud server having the ciphertext data and search token perform the searching operation and send back the search result to the requested data user. Another technique is known as keyword-based searchable encryption (KSE) [3] was also devised to perform the searching operation on encrypted outsourced data. Various types of models are currently available single-owner/single-user, also known as symmetric, multi-owner/single-user, and multi-owner/multi-user. Many symmetric searchable encryption (SSE) has been explored and have more properties such as data updating [4] search result verification [5], forward/backward privacy etc. Public key encryption that supports keyword searching has been explored to perform searching operations and enrich the functionality such as conjunctive, range, and subset search [6].

However, adopting the above-stated searchable encryption mechanisms, the data owners can not implement an effective access control mechanism, an essential element for every real application. To achieve both searching operation and access control simultaneously, researchers exploited the properties of attribute-based encryption (ABE) [7] technique. ABE’s access policy is an important factor, depending on how the data owner designs the access control policy during encryption. The ABE has two types which are: key-policy (KP-ABE) [8] and ciphertext-policy (CP-ABE) [9]. Using KP-ABE, the secret key is embedded inside the access policy, and data are encrypted according to user-specified attributes. Any user can decrypt if the access policy is his key matched the attribute set inside ciphertext data. While using CP-ABE, the access policy is associated with ciphertext, and the user secret key is attached with his attributes. Provided that the user attributes to meet the specified access policy, decryption can be performed successfully.

In short, the searching operation over encrypted data is an effective method to achieve privacy and confidentiality of outsourced data. Many schemes in the existing literature are available to achieve searchable encryption and access control. [10] in this paper, the author adopted only AND gate policy for access structure, authors in [11, 12] leveraged the Linear Secret Sharing Scheme (LSSS), used matrix as an access policy and based on AND, OR gates. Other schemes [13, 14] are based on bilinear pairing with a composite group order. However, the searching cost of the scheme is impractical. Most of the schemes mentioned above based on LSSS in which polynomial interpolation is used on the decryption side for reconstructing the shared secret are not efficient and flexible for resource constraint devices.

Efficiency related schemes for ABKS can broadly be categorized into:

### Outsourcing schemes

Most encryption and decryption operations are outsourced to resource-rich cloud service providers to reduce the computation overhead in these schemes [15–17]. As a result, the end-users perform a less or constant number of operations. This outsourcing can be performed either for encryption or decryption or both at the same time. However, these schemes strictly depend on the underlying framework and can not be applied to all the ABKS schemes.

### Online/offline schemes

The generation of index keyword and query token are divided into two phases [18, 19]. Most computations are done for index or token preparation during the first phase before knowing the exact specifics. So, when the specifics become known in the second phase, it rapidly assembles an intermediate index or token. The problem with these schemes is, the overall computation remains the same for the end-users.

### Non-pairing schemes

As the name suggests, these schemes avoid the most expensive and time-consuming bilinear operation with the lighter one, i.e., Elliptic Curve Cryptography (ECC) based scalar point multiplication operation [20, 21]. However, these schemes also suffer from the underlying linearity problem of attribute-based encryption.

Our proposed scheme avoids the expensive bilinear pairing operation and costly Lagrange interpolation for secret reconstruction simultaneously for the searching and decryption phase. Our main contribution made in this paper can be listed as follows:

1. The proposed scheme avoids costly bilinear pairing operation in the search phase and is free from complex Lagrange interpolation for secret reconstruction at the data user side.
2. Our proposed scheme supports the updation of access control policy without the liability of complete re-encryption of already stored ciphertext on the cloud.
3. The scheme also avoids the linear secret sharing scheme (LSSS) matrix and access tree construction to generate data user’s secret key components from their claimed set of attributes.
4. The security proof is given in the selective-set model under the Decisional Bilinear Diffie-Hellmen (DBDH)assumption and found to be collision-free.
5. The detailed experimental and informal analysis demonstrates the efficacy in terms of both communication and computation.

## Related work

For the first time, Song et al. [22] introduced searchable encryption where the data owner outsources the encrypted data to a remote storage server along with encrypted keywords. To search for a specific keyword data user sends information regarding the specified keyword. Based on this information, the storage server returns the requested results. A large number of schemes based on attribute-based encryption were proposed in the literature [23–26]. These ABE schemes were adopted to construct attribute-based keyword searching schemes. The access policy determining who can perform the decryption operation was used to decide who can perform the search operation. Zheng et al. [27] were the first to propose the scheme based on ABKS. They adopted both variants of ABE: KP and CP to construct the SE scheme. The scheme also has support for the verifiability of the search result performed by the cloud server. Later on, the scheme proposed by Lv Z et al. [14] had support for the revocation of the user. However, the number of pairing operations and secret key size were directly proportional to the number of attributes associated with the access policy. As a result, these schemes incur substantial computational overhead.

Wang et al. [15] introduce delegation in which a cloud server carried out the extensive computation task to address substantial computation. As a result of delegation, the architecture became complicated, and other third parties were involved in the system. Wang et al. [17] presented a scheme requiring a fixed number of pairing operations that support fast searching operations, but the size of the secret key was directly proportional to the number of attributes involved in the system. Zheng et al. [27] presented that big data mobile healthcare networks also support the verifiability of the search result. Hence, the contemporary approaches to ABKS heavily rely on the complex secret sharing mechanism of Lagrange interpolation and costly bilinear pairing operations. Our proposed scheme aims to achieve computational efficiency, decrease the key generation time, encryption, and decryption to make it flexible for devices with low processing and storage capabilities.

## Preliminaries

This section gives background knowledge about the bilinear map, access structure, and linear secret sharing scheme (LSSS).

### Bilinear map

Consider three multiplicative cyclic groups $G_{1}$, $G_{2}$ and $G_{T}$ having prime order *p*, where *P*, *Q* are generators of $G_{1}$ and $G_{T}$ respectively. $e:G_{1}\times G_{2}\to G_{T}$ is the bilinear map if it has below given properties:

1. *Bilinear:*
  -
$\forall P,Q\in G_{1}$ , and $\forall x,y\in {\mathbb{Z}}_{p}^{*}$, *e*(*xP*, *yQ*) = *e*(*P*, *Q*)^*xy*^
2. *Non-degenerate:*
  -
$\forall P,Q\in G_{1},e(P,Q)\neq 1$ .
3. *Commutable:*
  -
$\forall P,Q\in G_{1}$ , there must exist an algorithm to efficiently compute *e*(*P*, *Q*).

### Access structure

- Monotone access structure: if S is a set of attributes satisfying an access structure *T*, then any $S^{\prime }$ such that $S\subset S^{\prime }$ also satisfies T. For example, let say *T* = *X* ∩ *Y*, then both S={X,Y} and $S^{\prime }=\{X,Y,Z\}$ satisfy T.
- Non-monotone access structure: there exists $S^{\prime }$ in such a way $S\subset S^{\prime }$ does not satisfy *T*. For example, let say *T* = *S* ⊂⌝*Z*. Then in the previous example, only S satisfies T.

### Replicated secret sharing

The modular addition scheme [28], a special case of replicated secret sharing, a dealer can split a secret s into *k* shares and when all the shares combined, only then they can reconstruct the secret s. Sharing a secret s, where {*s*|*s* ∈ [0, *p* − 1]} for some integer *p*, the dealer randomly selects *k* − 1 values for *s*_*i*_ such that {*s*_*i*_|*i* ∈ [0, *k* − 1]} and computes $s_{k}=s-\sum_{i=1}^{k-1}\;mod\;p$. Share *s*_*i*_, where {*i*|*i* ∈ [1, *k* − 1]} are communicated to party *p*_*i*_. The original secret *S* can only be constructed by $s=\sum_{i=1}^{k}s_{i}$, hence only the dealer knows the secret *s* and other parties do not have any information regarding the secret.

## System model and security definitions

### System model

**Fig 2:**
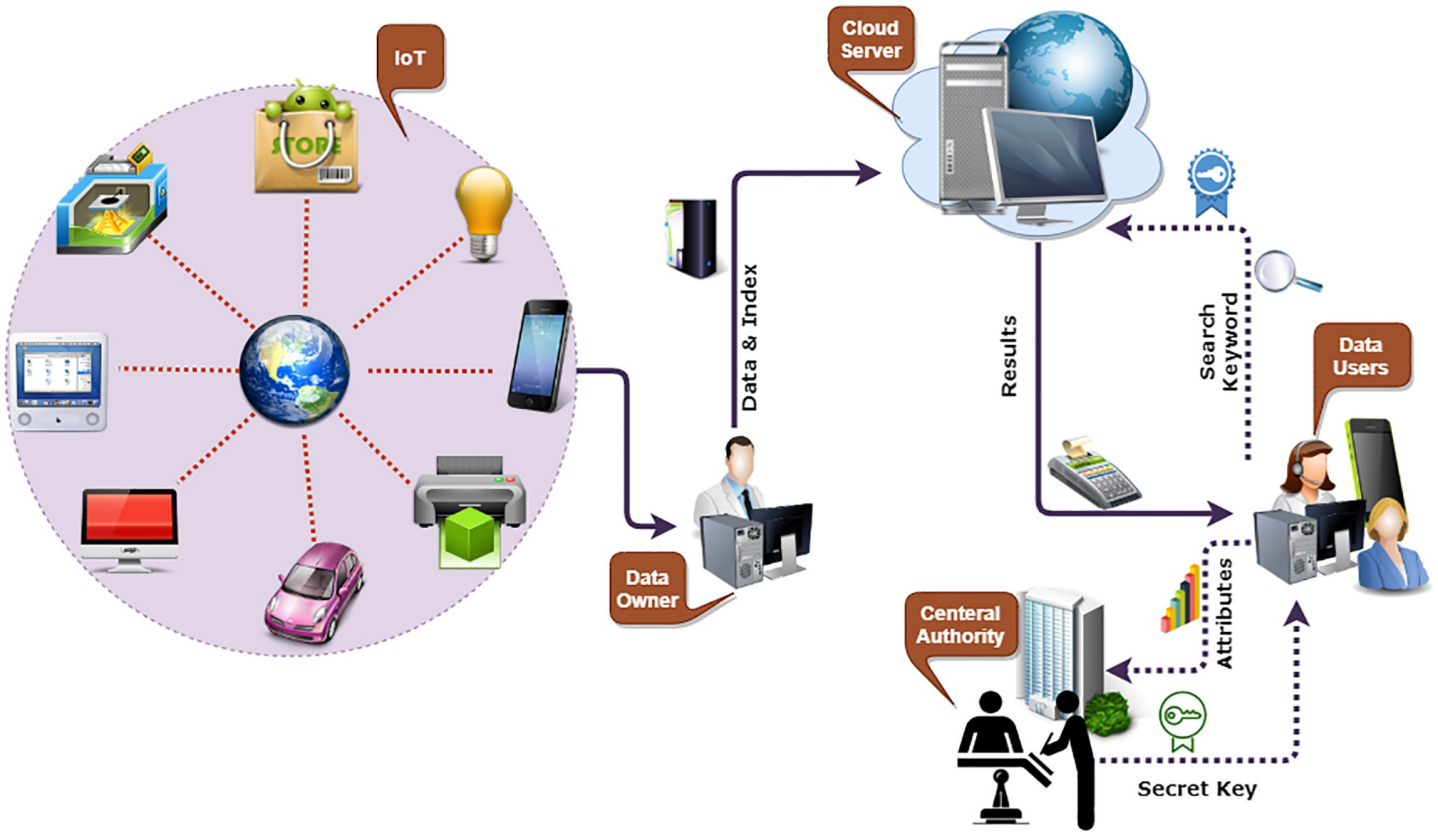
Proposed system overview.

Here we present the proposed system model. Specifically, there are four entities involved in the proposed system architecture, namely: Cloud Server (CS), Central Authority (CA), Data Owner (DO), and Data User (DU). As depicted in Fig 2.

1. **Central Authority**: As shown in Fig 2, we consider a central authority (CA) to be a trusted party responsible for initializing the whole system, generating the system parameters, and distributing secret keys.
2. **Cloud Server**: In the proposed system model, the cloud server provides storage resources. Upon receiving the authorized token from the data user (DU), it performs the searching operation and sends the DU search result. Cloud servers perform the search operation without knowing any information about the encrypted token and search result.
3. **Data Owner**: The data owner (DO) can be those who are willing to outsource their encrypted data to the cloud server. The DO encrypts the data according to the access control policy of his choice.
4. **Data User**: The data user (DU) are those who want to search over encrypted data. The DU executes the proposed *TokGen* algorithm to generate a search token for his interesting keywords and get the desired results.

Additionally, in our threat model, we consider the CS the “curious but honest” entity [29]. Most of the contemporary approaches to security also deploy this assumption. CA, DU, and DO are assumed to be fully honest and trusted entities.

### Security definition

The ABKS schemes require that the encryption algorithms not reveal any underlying keyword information in the index keyword and query token to an adversary. Thus, the privacy of the DO and DU should be maintained while outsourcing their respective data. The following security definitions are given to evaluate the security requirements between adversary A and challenger C in the form of a security game.

**Definition 1:** Our EFG-KSS scheme protects the index keyword from recovery attack in the chosen Plaintext Attack (CPA) model.

At the start of the game, the challenger publishes the public parameters to A. Adversary A selects challenge access tree $T^{*}$ and submits it to the C. A repeatedly asks for private key components of attribute set *S*_*j*_ = {*att*_*j*_ ∣ *att*_*j*_ ∈ *U*} and the encrypted index keyword ${ℐ}_{k_{1}},{ℐ}_{k_{1}},\ldots ,{ℐ}_{k_{n}}$ of keywords *k*_1_, *k*_2_, …, *k*_*n*_ such that non of the attribute set satisfies $T^{*}$. A submits two keywords *w*_*o*_ and *w*_1_ to C. Based on the outcome of flipping a fair binary coin *v* = {0, 1}, C encrypt *w*_*b*_ to get the index keywords. A adaptability submits an attribute sets *s*_*j*+1_, *s*_*j*+2_… to get its corresponding private key components $K_{A}^{s_{j+1}},K_{A}^{s_{j+2}}\ldots$ respectively, and the ciphertext $C_{j_{m+1}},C_{j_{m+2}}\ldots$ of keywords *k*_*j*+1_, *k*_*j*+2_… while none of these attribute set satisfies $T^{*}$. Finally, A output its guess *b*′ of b. The winning advantage of A is defined as $Adv_{A}$ = $|Pr(b=b^{\prime })-\frac{1}{2}|$. Now, if *Adv*_*A*_ is negligible, we would confirm that our scheme protect the index keyword from recovering attack in the chosen plaintext model.

**Definition 2:** Our query trapdoor algorithm protects the query token from recovery attack in the eavesdrop attack model.

A submits multiple queries for different keywords *q*_1_, *q*_2_, …, *q*_*n*_, In response to each A query, C outputs the ciphertext and sends it to A. A submits two query keywords *q*_0_ and *q*_1_ to C, which has not been queried earlier. C randomly selects a bit *b* ∈ {0, 1} and output *q*_*b*_, and submits it to A. A is allowed to ask for any number of queries, except that the query keyword *q*_0_ and *q*_1_ have not been queried before. Finally, A output its guess *b*′ of b. The winning advantage of A is defined as $Adv_{A}$ = $|Pr(b=b^{\prime })-\frac{1}{2}|$. Now, if *Adv*_*A*_ is negligible, we would confirm that our scheme protect the query token from recovery attack in the eavesdrop attack model.

**Definition 3:** Our proposed scheme ensures that if any of the compromised users is unable to decrypt a ciphertext individually, they are still unable to succeed to decrypt it by combining more than one secret key component or attribute.

## Proposed scheme

The following algorithms constitute the complete working mechanism of our proposed EFG-KSS scheme.

**Setup (λ):** Run by the CA for the initialization of the whole system, this algorithm proceed as follows:

a) Generate a bilinear map $\hat{e}:G_{1}\times G_{2}\to G_{T}$, where $G_{1}$, $G_{2}$ and $G_{T}$ are three multiplicative cyclic groups of prime order *P*, *g* is a generator of $G_{1}$.
b) Select a secure hash function $H:G_{1}\to \{0,1\}$.
c) For some integer *n*, generate the universal set of attribute *U* = {*att*_1_, *att*_2_…*att*_|*u*|_}. For each *att*_*i*_ ∈ *U*, select random elements *t*_1_, *t*_2_, …, *t*_*n*_ and $\alpha ,b\in {\mathbb{Z}}_{p}$.
d) Compute *Y* = *g*^*α*^, *y* = *e*(*g*, *g*)^*α*^, *g*^*b*^ and {*T*_*i*_ = *g*^*ti*^|*i* ∈ [1, *n*]}.
e) Set the public key as: *PK* = (*e*, *g*, *g*^*b*^, *y*, *Y*, {*T*_*i*_ = *g*^*ti*^|*i* ∈ [1, *n*]}), and the master key is *MK* = (*α*, {*t*_*i*_|*i* ∈ [1, *n*]}).

**KeyGen (*S*, *MK*):** This algorithm is run by *CA* to generate secret keys for authorized *DU*. On input the registered *DU* attribute set *S* = {*att*_1_, *att*_2_…*att*_|*m*|_} ⊂ *U*, this algorithm performs the following steps:

a) Select random values $R_{i}\in {\mathbb{Z}}_{p}^{*}$ such that $\left(t_{i}\times \sum_{i=1}^{\left|S\right|}R_{i})=(\sum_{i=1}^{\left|S\right|}t_{i}\times \alpha \right)$, and computes *do*

$=\frac{\sum_{i=1}^{\left|S\right|}R_{i}}{\sum_{i=1}^{\left|S\right|}t_{i}}$ .
b) Choose a random number $r\in {\mathbb{Z}}_{p}^{*}$ and computes *d*_1_ = *g*^*α*−*r*^.
c) For each *att*_*i*_ ∈ *S*, compute $d_{i}=g^{rt_{i}^{-1}}$.
d) Return the secret key $SK=(d_{o},d_{1},\{d_{i}=g^{rt_{i}^{-1}}|i\in [1,m]\})$.

**EncInd** (K,T,PK): This algorithm is executed by *DO* to encrypt a randomly chosen key $K\in {\mathbb{Z}}_{p}^{*}$ using access control structure T of his choice in the from of boolean formula. On input the public key *PK* of *CA*, the *DO* performs the following steps:

a) Chooses a random secret *s* and $r\in {\mathbb{Z}}_{p}^{*}$ and computes *A*_*verf*_ = (*Y*)^*s*^ = (*g*^*α*^)^*s*^, *C*_0_ = *g*^*s*^ and $C_{1}=K.y^{s}=K.e(g,g{)}^{\alpha s}$.
b) Given the access tree T, the algorithm performs the following steps to distribute secret *s* according to node *v* in a top-down approach:
  1) if the root node is *v*, (*i*.*e*., *v* = *root*), set its value to *s*.
  2) Recursively, for each inner node (including the root node), do the following:
  2.1) if the inner node *v* represents the *AND* gate, for each of its child node excluding the last child, set its value to *s*_*i*_ where *s* ∈ [1, *p* − 1], and set the value of its last child to $s_{n}=s-\sum_{i=1}^{n-1}s_{n}\text{mod}\;\text{p}$
  2.2) if the inner node *v* represents the *OR* gate, it sets every child node value to its parent node value.
  3) For each attribute *a*_*j*,*i*_ attached to leaf node ∈T, compute $C_{j,i}=T_{j}^{si}$.
  4) Compute and set ${ℐ}_{w}=e(g^{H(w).s},g^{\alpha }).e(g,g{)}^{\alpha .s}$, ${ℐ}_{w}^{\prime }=g^{s}$
  5) Set the cipher-text $CT=(T,A_{verf},{ℐ}_{w},{ℐ}_{w}^{\prime },C_{0},$
    *C*_1_, {*C*_*j*,*i*_|*a*_*j*,*i*_ ∈ *τ*})

**TokenGen** (SK,q): This algorithm is run by *DU* to generates token for its interested keywords *q*.

a) The DU compute *tok*_1_ = *g*^*α*.*H*(*q*)^ and *tok*_2_ = *g*^*α*^ and set $T_{\left(q\right)}=(tok_{1},tok_{2},d_{0})$

**Search** ($ℐ,T_{q}$): This algorithm is run by *CS* to securely perform the search operation over outsourced encrypted index according to the query token submitted by the *DU*. By secure, we mean that the stored data elements in the index token or the encrypted data itself reveal no information to the *CS* after completion of the search operation. By running this algorithm, the *CS* needs to find out if this *DU* possess the attributes corresponding to each leaf node of T, s⊨T and also check out if it has the stored index ${ℐ}_{w}$ equal to the query token $T_{q}$, *w* = *q*. More specifically, this algorithm returns 1 if and only if the below two conditions hold simultaneously:

a) Access confirmation: Taking $\left\{C_{j,i}|a_{j,i}\in T\right\}$ from *CT* and *d*_0_ from T(q), this process needs to compute $A_{verf}^{\prime }=\prod_{i\in s}(C_{\left(j,i\right)}{)}^{d_{0}}$. After which the *CS* can find out whether it equals the *A*_*verf*_ in the ciphertext *CT* set by *DO*.
b) Token confirmation: After ciphertext *CT* is accessible, i.e., S⊨T, the *CS* needs to find out whether index keyword *w* is equal to the query token *q*, *w* = *q*, by evaluating the validity of equation $e(\tau_{w}^{\prime },tok_{1})\times e(\tau_{w}^{\prime },tok_{2})$, otherwise the algorithm returns ⊥ to the DU.

**Dec** (SK,CT): This algorithm is run by *DU* to retrieve the symmetric key K, uses to decrypt $D_{∥}(Data)$ the outsourced encrypted data. This algorithm proceeds as follows:

a) The algorithm select the smallest set *S*′ ⊆ *S* that satisfies T.
b) For each *a*_*i*_ ∈ *S*′, compute
  $$\begin{array}{ll} & \prod_{a_{i}\in S^{\prime }}e(C_{i},d_{i})\\ = & \prod_{a_{i}\in S^{\prime }}e(T_{i}^{si},g^{rt_{i}^{-1}})\\ = & \prod_{a_{i}\in S^{\prime }}e(g^{t_{i}s_{i}},g^{rt_{i}^{-1}})\\ = & e(g,g{)}^{rs}. \end{array}$$
c) Then compute
  $$\begin{array}{ll} & e(C_{0},d_{1}).e{\left(g,g\right)}^{rs}\\ = & e(g^{s},g^{\alpha -r}).e{\left(g,g\right)}^{rs}\\ = & e{\left(g,g\right)}^{s\alpha }. \end{array}$$
d) Finally derive the symmetric key K as
  $$\begin{array}{ll} & \frac{C_{1}}{e{\left(g,g\right)}^{\alpha .s}}\\ = & \frac{K.e{\left(g,g\right)}^{\alpha .s}}{e{\left(g,g\right)}^{\alpha s}}\\ = & K. \end{array}$$

## EFG-KSS analysis

This section presents a detailed analysis of our scheme’s correctness, complexity, access control policy update, and security proof.

### Correctness analysis

First of all, *CS* needs to confirm whether *DU*’s set of attributes *S* meets the access control policy set by the *DO*. In other words, the *CS* ensures the access authorization request of *DU* for the *DO* outsourced index keyword *w*. As we know from Algorithm *EncInd*, the *DO* computes and set the access verification to

$$\begin{array}{c} A_{verf}={\left(Y\right)}^{s}={\left(g^{\alpha }\right)}^{s}=g^{\alpha .s} \end{array}$$ (1)

Hence, the *CS* need to compute ∏_*i*∈*s*_(*C*_*j*,*i*_)^*do*^, to find out whether it output the same value as required by the *DO* in its ciphertext.

$$\begin{array}{ll} A_{verf} & =\prod_{i\in S}{\left(C_{j,i}\right)}^{do}\\ RHS & =\prod_{i\in S}{\left(C_{j,i}\right)}^{do}\\ & =\prod_{i\in S}[{\left(T_{j}\right)}^{si}{]}^{do}\\ & =\prod_{i\in S}[{\left(g^{t_{i}^{\left(s_{i}\right)}}\right]}^{do}\\ & =\prod_{i\in S}[{\left(g\right)}^{t_{i}\times s_{i}}{]}^{do}\\ & ={\left[g^{\sum_{i\in S}\left(t_{i}\times s_{i}\right)}\right]}^{do}\\ & ={\left[g^{\sum_{i=1}^{s}t_{i}\times \sum_{i=1}^{s}s_{i}}\right]}^{\frac{\sum_{i=1}^{s}R_{i}}{\sum_{i=1}^{s}t_{i}}}\\ & =g^{\sum_{i=1}^{s}s_{i}\times \sum_{i=1}^{s}R_{i}}\\ & =g^{s.\alpha }\\ & =A_{verf}=LHS \end{array}$$

In case of successful access authorization, the *CS* further needs to confirm the similarity between the keyword in the form of submitted query token $T_{w}$ against the stored index ${ℐ}_{w}$ keyword by evaluating the following equation validity.

$$\begin{array}{ll} I_{w} & =e(I_{w}^{\prime },tok_{1})\times e(I_{w}^{\prime },tok_{2})\\ R.H.S & =e(I_{w}^{\prime },tok_{1})\times e(I_{w}^{\prime },tok_{2})\\ & =(g^{s},g^{\alpha H(q)})\times (g^{s},g^{\alpha })\\ & =e{\left(g,g\right)}^{\alpha .s.H(q)}\times e{\left(g,g\right)}^{\alpha .s}\\ L.H.S & =I_{w}\\ & =e(g^{s.H(w)},g^{\alpha })\times e{\left(g,g\right)}^{\alpha .s}\\ & =e{\left(g,g\right)}^{\alpha .s.H(w)}\times e{\left(g,g\right)}^{\alpha .s}\\ & {\underline{\underline{w=q}}}_{R.H.S}. \end{array}$$

### Complexity analysis

This section presents a theoretical analysis in terms of time complexity by comparing our proposed scheme with the schemes of CP-ABKS [27], and CP-ABSE [30]. Both of these schemes provide a convincing performance comparison with our proposed scheme. The notations used for this comparison are shown in Table 1.

**Table 1:** Notations.

Notations	Description
*P*	Bilinear pairing
$E_{G_{1}}$	Exponentiation in $G_{1}$
$M_{G1}$	Multiplication in $G_{1}$
$M_{G_{2}}$	Multiplication in $G_{2}$
$E_{G_{2}}$	Exponentiation in $G_{2}$
$M_{G_{q}^{*}}$	Multiplication in ${\mathbb{Z}}_{q}^{*}$
∣*X*∣	Elements in set *X*
∥*y*∥	Length of element *Y* in bits
${\mathbb{Z}}_{q}^{*}$	Element in ${\mathbb{Z}}_{q}^{*}$
*H*	Compute hash $H:\{0,1{\}}^{*}\to {\mathbb{Z}}_{q}^{*}$
H*	Compute hash $H:\{0,1{\}}^{*}\to G_{1}$
*S*	Attribute set of a DU.
*T*	Selective attribute set of DU
*N*	Minimum attribute set for access structure.

Computation and output overhead of each algorithm for EFG-KSS, CP-ABSE, and CP-ABKS are shown in Tables 2–4, respectively. Here, we do not consider an operation like a basic arithmetic operation; multiplication, addition, and subtraction in ${\mathbb{Z}}^{*}$, hash function because of its less time consumption. We also do not consider the computation cost incur due to the successful search query. As a result, the search output size is set to zero for all the schemes.

**Table 2:** Computation and output cost of our scheme.

Algorithm	Computation	Output
Setup	$P+|U|E_{G_{1}}+Z_{q}^{*}$	2∣*U*∣∥*G*_1_ ∥ + ∥ *G*_2_∥
KeyGen	$|S|E_{G_{1}}+(|S|H)Z_{q}^{*}$	(∣*S*∣ + 2)∥*G*_1_∥
EncInc	$(|T|+3)E_{G_{1}}+P+H+Z_{q}^{*}$	(∣*T*∣ + 2)∥*G*_1_ ∥ + ∥ *G*_2_∥
TokGen	$E_{G_{1}}+H$	$|X||Z_{q}^{*}|+2∥G_{1}∥$
Search	$|N|E_{G_{2}}+4P$	0

**Table 3:** Computation and output cost of CP-ABSE in [30].

Algorithm	Computation	Output
Setup	$P+2E_{G_{1}}+2{\mathbb{Z}}_{q}^{*}$	$|{\mathbb{Z}}_{q}^{*}|+3∥G_{1}∥∥G_{2}∥$
KeyGen	$|S|H^{\prime }+(|S|+1){\mathbb{Z}}_{q}^{*}+(2|S|+4)E_{G_{1}}$	$(2|S|+2)∥G_{1}∥$
EncInd	$(2|T|+2)E_{G_{1}}+P+{\mathbb{Z}}_{q}^{*}+H+E_{G_{2}}+|T|H^{\prime }$	$(2|T|+1)∥G_{1}∥+∥G_{2}∥$
TokenGen	$E_{G_{1}}+|S|M_{G_{1}}+H$	$(2|S|+1)∥G_{1}∥$
Search	$(2|\mathbb{N}|+1)P+|\mathbb{N}|E_{G_{2}}+(|\mathbb{N}|+1)M_{G_{2}}$	0

**Table 4:** Computation and output cost of of CP-ABKS in [27].

Algorithm	Computation	Output
Setup	$3E_{G_{1}}+3{\mathbb{Z}}_{q}^{*}$	$3|{\mathbb{Z}}_{q}^{*}|+4∥G_{1}∥$
KeyGen	$(|S|+1){\mathbb{Z}}_{q}^{*}+(3|S|+1)E_{G_{1}}+|S|H^{\prime }+|S|M_{G_{1}}$	$(2|S|+1)∥G_{1}∥$
EncInd	$2{\mathbb{Z}}_{q}^{*}+(2|X|+6)E_{G_{1}}+H+M_{G_{1}}+|X|H^{\prime }$	$(2|X|+3)∥G_{1}∥$
TokenGen	$H+M_{G_{1}}+(2|S|+4)E_{G_{1}}$	$(2|S|+3)∥G_{1}∥$
Search	$(2|\mathbb{N}|+3)P+(|\mathbb{N}|+2)M_{G_{2}}+(|\mathbb{N}|)E_{G_{2}}$	0

From Table 2, we observe that our scheme suffers from high storage and computation cost for the *Setup*phase than both the scheme CP-ABKS and CP-ABSE. However, the *Setup* phase runs on the resource-rich trusted authority and one-time operation, making it acceptable in real-world scenarios and resource-scarce devices.

From Tables 3 and 4, we can notice that EFG-KSS outperforms both the CP-ABSE and CP-ABKS on the KeyGen, EncInd, TokenGen, and Search algorithm complexity because of less exponentiation and pairing operation requirements.

### Access control policy update

**Fig 3:**
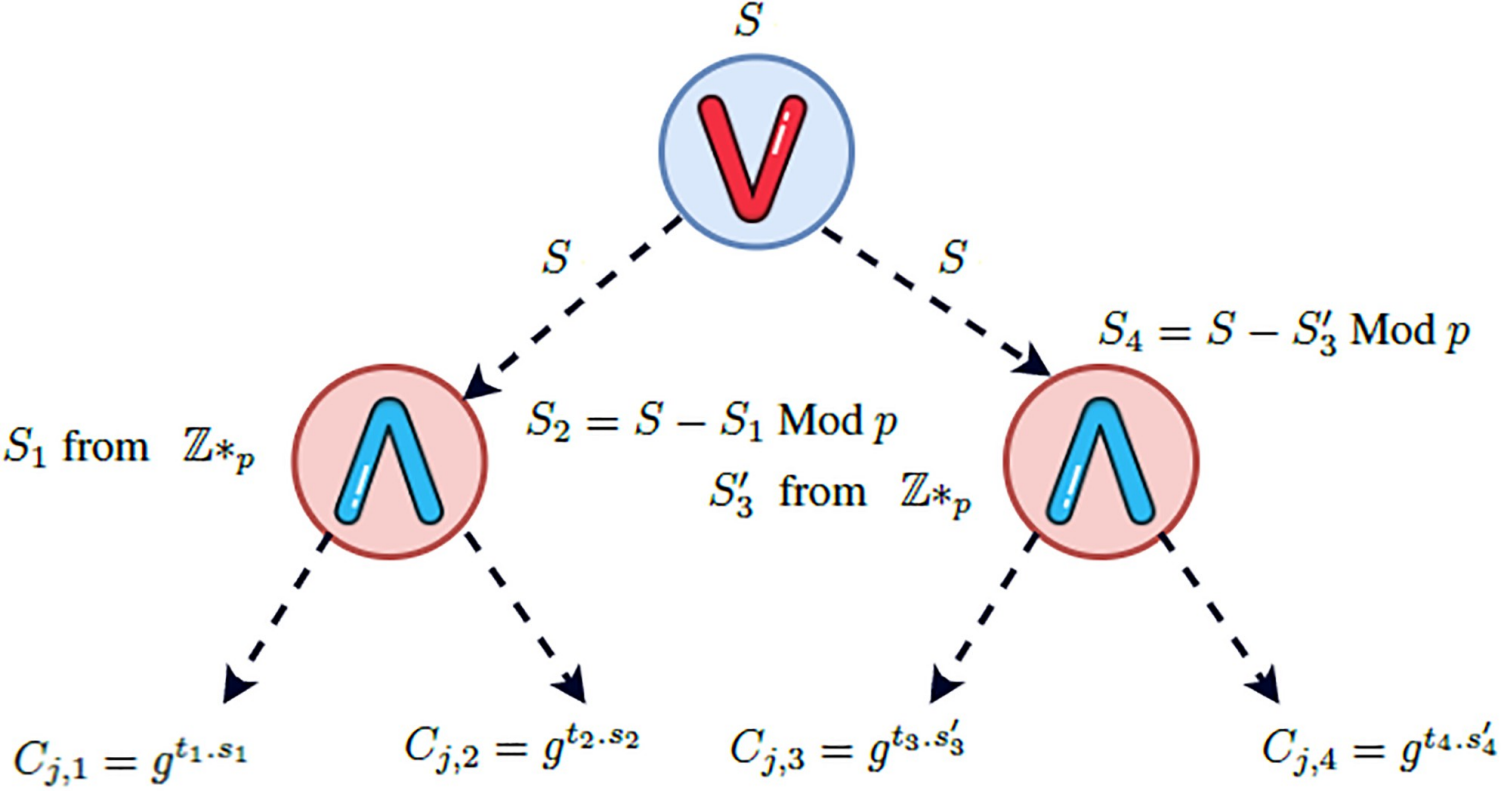
Access policy for T.

**Fig 4:**
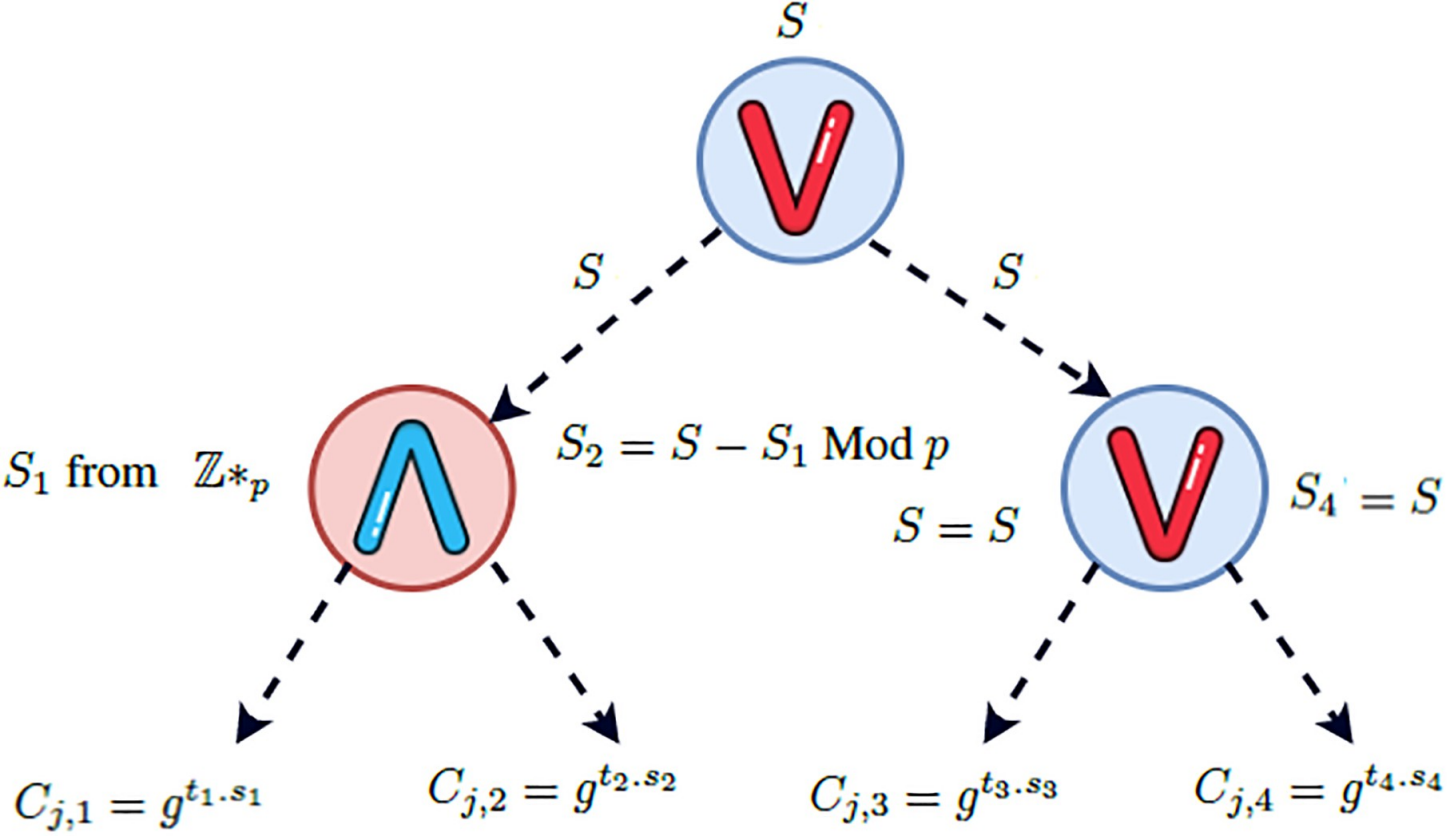
Access policy for $T^{\prime }$.

In EFG-KSS scheme, the data owner do not need to entirely re-encrypt the ciphertext in case of his access control policy updation. Our scheme utilizes access tree T as access control policy. Let a data user wants to update his already defined access control policy from $T=(T_{1}\wedge T_{2})\vee (T_{3}\wedge T_{4})$, shown in Fig 3. To a newly defined access control policy $T^{\prime }=(T_{1}\wedge T_{2})\vee (T_{3}\vee T_{4})$, shown in Fig 4.

Recall from EncInd algorithm, to encrypt a symmetric key K, this algorithm in its first phase select a random number $s\in {\mathbb{Z}}_{p}^{*}$, compute *C*_*o*_ = *g*^*s*^ and $C_{1}=K.y^{s}=K.e(g,g{)}^{\alpha .s}$.

Since only the second phase encryption is based on some access control policy T, the Algorithm compute: $\left\{C_{j,1}=T_{1}^{s_{1}},C_{j,2}=T_{2}^{s_{2}},C_{j,3}=T_{3}^{s_{3}},C_{j,4}=T_{4}^{s_{4}}|j\in T\right\}$. The final ciphertext is set to $C_{T}=(T,C_{0},C_{1}\{C_{j,1},C_{j,2},C_{j,3},C_{j,4}|j\in T\})$

To change the access control policy from T to $T^{\prime }$, we do not need to re-encrypt the first phase encryption since the access control policy T is enforced only by the second phase encryption. Furthermore, during the second phase, we need to update the ciphertext components *C*_*j*,3_ and *C*_*j*,4_ only.

Hence, the updated ciphertext elements are:

$$\left\{C_{j,1}=T_{1}^{s_{1}},C_{j,2}=T_{2}^{s_{2}},C_{j,3}=T_{3}^{s_{3}^{\prime }},C_{j,4}=T_{4}^{s_{4}^{\prime }}|j\in T^{\prime }\right\}$$

As a result, the new ciphertext is $C_{T}=(T^{\prime },C_{0},C_{1}\{C_{j,1},C_{j,2},C_{j,3},C_{j,4}|j\in T^{\prime }\})$

### Security analysis

**Theorem 1:** Based on the DBDH hardness assumption, no probabilistic polynomial-time adversary (PPT) can break *EncInd* algorithm associated with index keyword encryption with a challenge access tree $T^{*}$.

**Proof:** If A can recover keyword information from EncInd algorithm in polynomial time T with non-negligible advantage *ε*, then we can construct an algorithm ℬ which can play Decisional-BDH game with non-negligible advantage $\frac{\varepsilon }{2}$. The challenger C at the start of the game setup random elements $a,b,c,z\in {\mathbb{Z}}_{q}^{*}$. C flips a fair binary coin *μ* ∈ {0, 1} and sets ${\mathbb{Z}}_{\mu }=e(g,g{)}^{abc}$, when *μ* = 0 and ${\mathbb{Z}}_{\mu }=e(g,g{)}^{z}$ if *μ* = 1. The challenger C gives ${\mathbb{Z}}_{\mu }$ to simulator ℬ. Both ℬ and adversary A play the game as follows:

**Init:** A selects challenge access tree $T^{\prime }$ and sends it to the simulator.

**Setup:** ℬ computes the public parameter $e(g,g{)}^{\alpha }=e(g,g{)}^{\bar{x}}.e(g,g{)}^{ab}$ by letting $\alpha =ab+\bar{x}$, where $\bar{x}\in {\mathbb{Z}}_{q}^{*}$. For all *att*_*j*_ ∈ *S*, ℬ checks whether $att_{j}\in T^{*}$, sets $T_{j}=g^{k_{j}}$ (here $t_{j}=\frac{b}{k_{j}}$) otherwise sets $T_{j}=B^{\frac{1}{kj}}$ (so here *t*_*j*_ = *k*_*j*_) where kj is a random element $\in {\mathbb{Z}}_{q}^{*}$. Finally sends the the public parameter to A.

**Phase 1:** A repeatedly asks for private key components of attribute set *S*_*j*_ = {*att*_*j*_ ∣ *att*_*j*_ ∈ *U*} and the encrypted index keyword ${ℐ}_{k_{1}},{ℐ}_{k_{1}},\ldots ,{ℐ}_{k_{n}}$ of keywords *k*_1_, *k*_2_, …, *k*_*n*_ such that non of the attribute set satisfies $T^{*}$.

Now ℬ selects $R_{i}\in {\mathbb{Z}}_{q}^{*}$ and set $d_{0}=\frac{R}{\sum_{1}^{\left|s_{j}\right|}att_{j}}$. Also these private keys must produce legal query trapdoor. The simulator ℬ sets $d_{1}=g^{\bar{x}-r^{\prime }jb}$ by letting

*r* = *ab* + *r*′*b*

$$\begin{array}{ll} r & =ab+r^{\prime }b\\ d_{1} & =g^{\bar{x}-r^{\prime }jb}\\ & =g^{\alpha -ab-r^{\prime }jb}\\ & =g^{\alpha -(ab+r^{\prime }jb)} \end{array}$$

The simulator ℬ for each *att*_*j*_ not in $T^{*}$, computes $d_{j}=g^{(ab+r^{\prime }b)k_{j}/b}$, since *t*_*j*_ = *b*/*k*_*j*_ and *r* = *ab* + *r*′*b*. Where for each *att*_*j*_ ∈ *S*_*j*_ at set the valid secret key component to $d_{j}=A^{k_{j}}g^{k_{j}r^{\prime }}$ and can be computed by the ℬ as:

$$\begin{array}{ll} d_{j} & =g^{(ab+r^{\prime }b)k_{j}/b}\\ & =g^{ak_{j}}g^{k_{j}r^{\prime }}\\ & =A^{k_{j}}g^{k_{j}r^{\prime }} \end{array}$$

Finally, the simulator ℬ sends the $SK_{s_{j}}=(d_{0},d_{1},\{d_{j}|j\in (1,n)\})$ to A.

**Challenge:** A encrypts two keywords *ω*_0_ and *ω*_1_ to generates the corresponding index keyword. Submit it along with access structure $T^{*}$ to ℬ. Based on the outcome of flipping a coin *V* = {0, 1}, the simulator ℬ output the ciphertext as follows:

$C_{w_{b}}^{*}=(T^{*},A_{vrf},C_{0},C_{1},\{{ℐ}_{w_{b}}=e(C^{H(w_{b})},g^{\left(ab+\bar{x}\right)}).{\mathbb{Z}}_{\mu }.e\left(g^{c},g^{\bar{x}})\}),\{{ℐ}_{w_{b}}^{\prime }=g^{c}\},\{C_{j,i}|j\in T^{*}\right\}$ . Finally, ℬ sends $C_{w_{b}}^{*}$ to A.

**Phase 2:** A adaptability sends an attribute sets *s*_*j*+1_, *s*_*j*+2_… to get its corresponding private key components $K_{A}^{s_{j+1}},K_{A}^{s_{j+2}}\ldots$ respectively, and the ciphertext $C_{j_{m+1}},C_{j_{m+2}}\ldots$ of keywords *K*_*j*+1_, *K*_*j*+2_… while none of these attribute set satisfies $T^{*}$.

**Guess:** A output its guess *b*′ of *b*. Since none of the attribute sets satisfies the $T^{*}$, A can not let the search algorithm to trivially decide *b* = 0 or *b* = 1. Therefore, A can use the index keyword ${ℐ}_{w_{b}}^{*}$ to recover keyword information to decide *b* = 0 or *b* = 1. The possibility for both the cases are given bellow:

- For ${\mathbb{Z}}_{\mu }=e(g,g{)}^{abc}$ we have *μ* = 0 and ${ℐ}_{w_{b}}^{*}=(T^{*},{ℐ}^{*}=e(g^{cH(w_{b})},g^{\left(ab+\bar{x}\right)}).e(g,g{)}^{abc})$

$e(g^{c},g^{\bar{x}}),$

$\left({ℐ}_{w_{b}}^{\prime }=g^{c}),\{C_{j,i}|i\in T^{*}\right\}$

Since s and *α* are randomly chosen for the index keyword generation, we let *c* = *s* and $(ab+\bar{x})=\alpha$, the ciphertext can be denoted as ${ℐ}_{w_{b}}^{*}=(T^{*},\{{ℐ}_{w_{b}}^{*}=e(g^{H(w).s}.g^{\alpha }).e(g,g{)}^{\alpha s}\},$

$\left\{{ℐ}_{w_{b}}^{*}=g^{s}\},\{C_{j,i}|j\in T\}\right)$

- If, then For ${\mathbb{Z}}_{\mu }=e(g,g{)}^{z}$, we have *μ* = 1 and the ciphertext is

${ℐ}_{w_{b}}^{*}=(T^{*},\{{ℐ}_{w_{b}}^{*}=e(g^{H(w).s}.g^{\alpha }).e(g,g{)}^{z}\},\{{ℐ}_{w_{b}}^{*}=g^{s}\},\{C_{j,i}|j\in T\})$

Since z is a randomly selected element, which also render ${ℐ}_{w_{b}}^{*}$ is a random looking element to an adversary A and hence reveal no information about *w*_*b*_. A output its guess *b*′ ∈ {0, 1}.

If *b*′ = *b*, ℬ output *μ*′*s* guess *μ*′ = 0 and ${\mathbb{Z}}_{\mu }=e(g,g{)}^{abc}$. When ${\mathbb{Z}}_{\mu }=e(g,g{)}^{abc}$, the challenger C sends a valid encryption parameter and ${ℐ}_{w_{b}}^{*}$ is a valid index keyword. Therefore, the advantage of an adversary A to recover *H*(*wb*) from ${ℐ}_{wb}^{*}$ is

$$\begin{array}{c} Pr\left[b^{\prime }=b|Z_{\mu }=e{\left(g,g\right)}^{abc}\right]=\frac{1}{2}+\varepsilon \end{array}$$

If *b*′ = *b*, ℬ output *μ*′*s* guess *μ*′ = 1 and ${\mathbb{Z}}_{\mu }=e(g,g{)}^{z}$. When ${\mathbb{Z}}_{\mu }=e(g,g{)}^{z}$, the challenger C sends a random encryption parameters and hence, ${ℐ}_{w_{b}}^{*}$ is not a valid index keyword. Therefore, A does not gain information about *H*(*w*_*b*_) from ${ℐ}_{w_{b}}^{*}$, hence we have

$$\begin{array}{c} Pr\left[b^{\prime }\neq b|Z_{\mu }=e{\left(g,g\right)}^{z}\right]=\frac{1}{2} \end{array}$$

The overall advantage of ℬ solving the DBDH problem is as follows:

$|\frac{1}{2}Pr[\mu^{\prime }=\mu |\mu =0]+\frac{1}{2}+Pr[\mu^{\prime }=\mu |\mu =1]-\frac{1}{2}|$

$=|[\frac{1}{2}(\frac{1}{2}+\varepsilon )+\frac{1}{2}.\frac{1}{2}]-\frac{1}{2}|$ $=\frac{\varepsilon }{2}$ .

**Theorem 2:** On the assumption of Discrete Logarithm (DL) problem, our proposed query keyword encryption is secure against token recoverable attack in eavesdropper security model.

**Proof:** Below security game between the the adversary A and challenger C is run to prove the above theorem.

**Phase 1:** A submits multiple query for different keywords *q*_1_, *q*_2_, …, *q*_*n*_, In response to each A query, C outputs the following ciphertext:

$$\begin{array}{c} T_{A(q_{i})}=\left(T_{1}=g^{\alpha H(q_{i})},T_{2}=g^{\alpha },do\right) \end{array}$$

**Challenge:** C receives two query keywords *q*_0_ and *q*_1_ from A, which have not queried earlier. C randomly selects a bit *b* ∈ {0, 1} and computes *q*_*b*_ as:

$$\begin{array}{c} T_{A(q_{b})}=\left(T_{1}=g^{\alpha H(q_{b})},T_{2}=g^{\alpha },do\right) \end{array}$$

and submit it to A.

**Phase 2:** A is allowed to sends further queries, except that the query keyword *q*_0_ and *q*_1_ have never been queried before.

**Guess:** A output its guess *b*′ of *b*. As A has no access to the encryption oracle and also without knowing *α*, it is not able to efficiently compute $T_{A(q_{1})}^{*}$ and $T_{A(q_{0})}^{*}$. Thus, as long as the DL assumption is intractable, the probability that A output the correct guess *b*′ = *b* is at most $\frac{1}{2}$.

**Theorem 3:** Our proposed scheme provides collision resistance under the Computational Diffie-Hellmen (CDH) assumption. If any of the compromised users cannot decrypt a ciphertext, they can still decrypt it by combining more than one secret key component or attribute.

**Proof:** Similar to other ABKS schemes, our proposed scheme also avoids the integration of secret keys or attributes, which is the most probable attack in the ABE scenario. More specifically, our proposed scheme considers the corruption of any data used as some overlapping individuals attribute among them may exist. For example, assume a data owner perform some encryption by associating an access control policy A=(student *AND* *university*) *OR* (*professor* *AND* *city*), to its ciphertext. Bob and Carl’s data users possess secret key against these attribute sets *S*_*B*_ = {*student*, *city*} and *S*_*C*_ = {*professor*, *carl*} respectively. Given their respective set of attributes, both the data user can not decrypt the ciphertext individually.

Now even if both the data users combine their secret key for the missing attribute, they should not decrypt the ciphertext encrypted under A. To avoid the collusion attack, the KeyGen algorithm of our proposed scheme randomly selects a value $r\in {\mathbb{Z}}_{p}^{*}$ and *R*_*i*_ for each user independently. Hence, the resultant secret key components can not be combined since they are generated randomly.

The secret key components of our proposed scheme are

$$\begin{array}{c} d_{o}=\frac{\sum_{i=i}^{s}R_{i}}{\sum_{i=1}^{s}t_{i}},d_{1}=g^{\alpha -r},\left\{d_{i}=g^{rt_{i}^{-1}}|i\in \left[1,n\right]\right\} \end{array}$$

Their individual *R*_*i*_ and *r* are randomly selected to meet the equation

$$\begin{array}{c} \sum_{i=1}^{|s|}\left(t_{i}R_{i}\right)=\alpha \times \sum_{i=1}^{|s|}t_{i} \end{array}$$

and to compute $d_{i}=g^{rt_{i}^{-1}}|i\in [1,s]$ respectively. Given the CDH assumption is hard, compromised data user will never be able to compute $\left(\alpha \times \sum_{i=1}^{|s|}t_{i}\right)$ and $g^{rt_{i}^{-1}}$ because of *R*_*i*_ and *r* from different data users.

## Performance analysis

To precisely evaluate and compare the performance of our proposed scheme with the two schemes mentioned above, this section presents experimental results for a series of experimental simulations. The experimental execution setting is Intel Core i5 Processor 2.4 GHz, 4GB RAM, on a Ubuntu 14. The implementation environment consists of a standard cryptographic Charm-Crypto library Version 0.42 with Spyder 2.2.5 IDE.

### Storage cost evaluation

**Fig 5:**
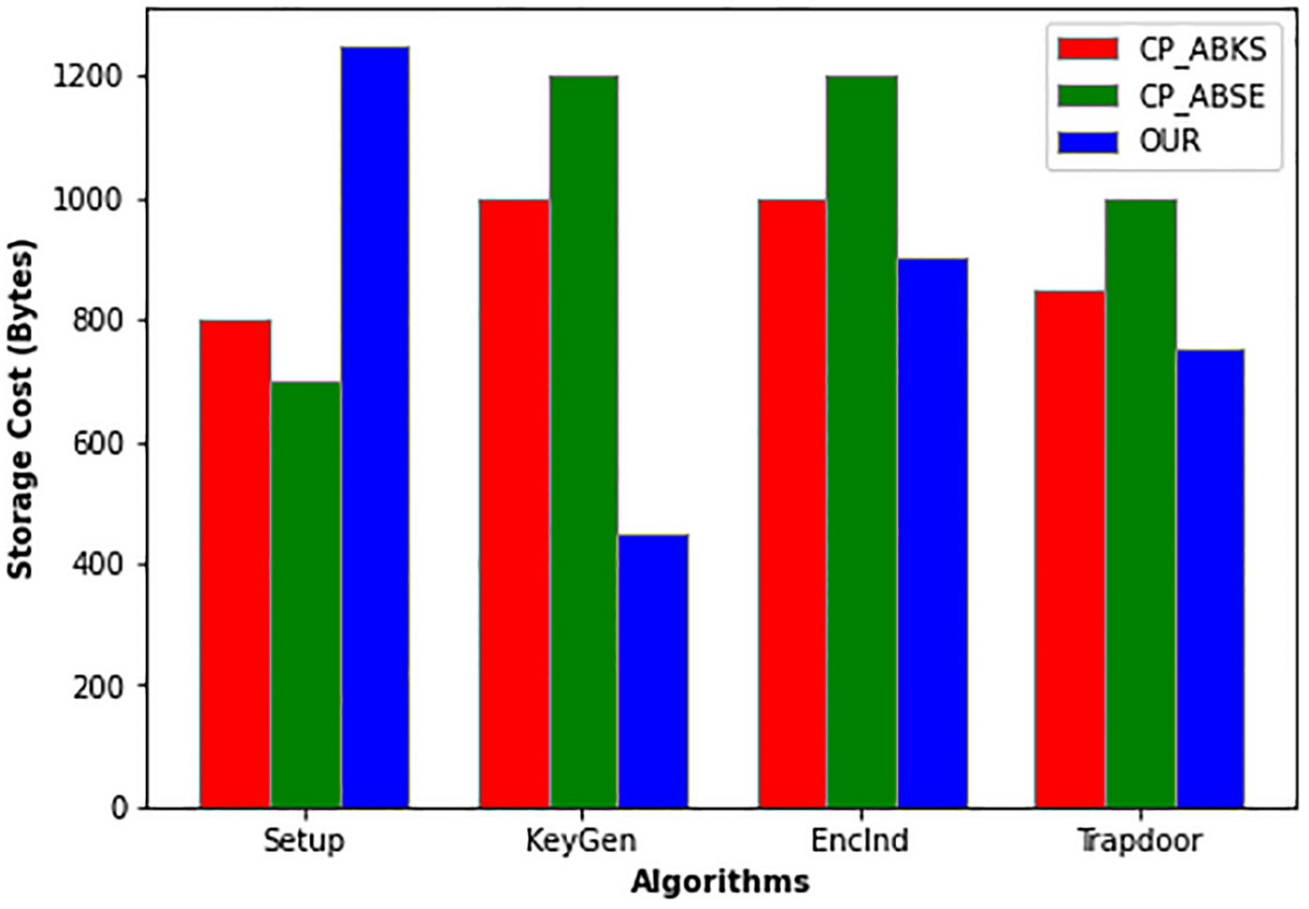
The storage cost of each algorithm.

For uniformity, in the experiment, we set ∣*X*∣ and ∣*S*∣ to be 10. Fig 5 depicts the storage cost of each algorithm in CP-ABSE, CP-ABKS, and EFG-KSS. Although our scheme yield higher storage cost when compared with other schemes for the *Setup* algorithm. In practice, this extra storage cost is acceptable; we know the *Setup* algorithm is run by a trusted attribute authority and is a one-time process. As evident from Fig 5 our proposed scheme takes less storage cost for *KeyGen*, *TokenGen*, and *EncInd*. Here the search algorithm space is ignored for its only output values 1 or 0.

### Evaluation of KeyGen algorithm

**Fig 6:**
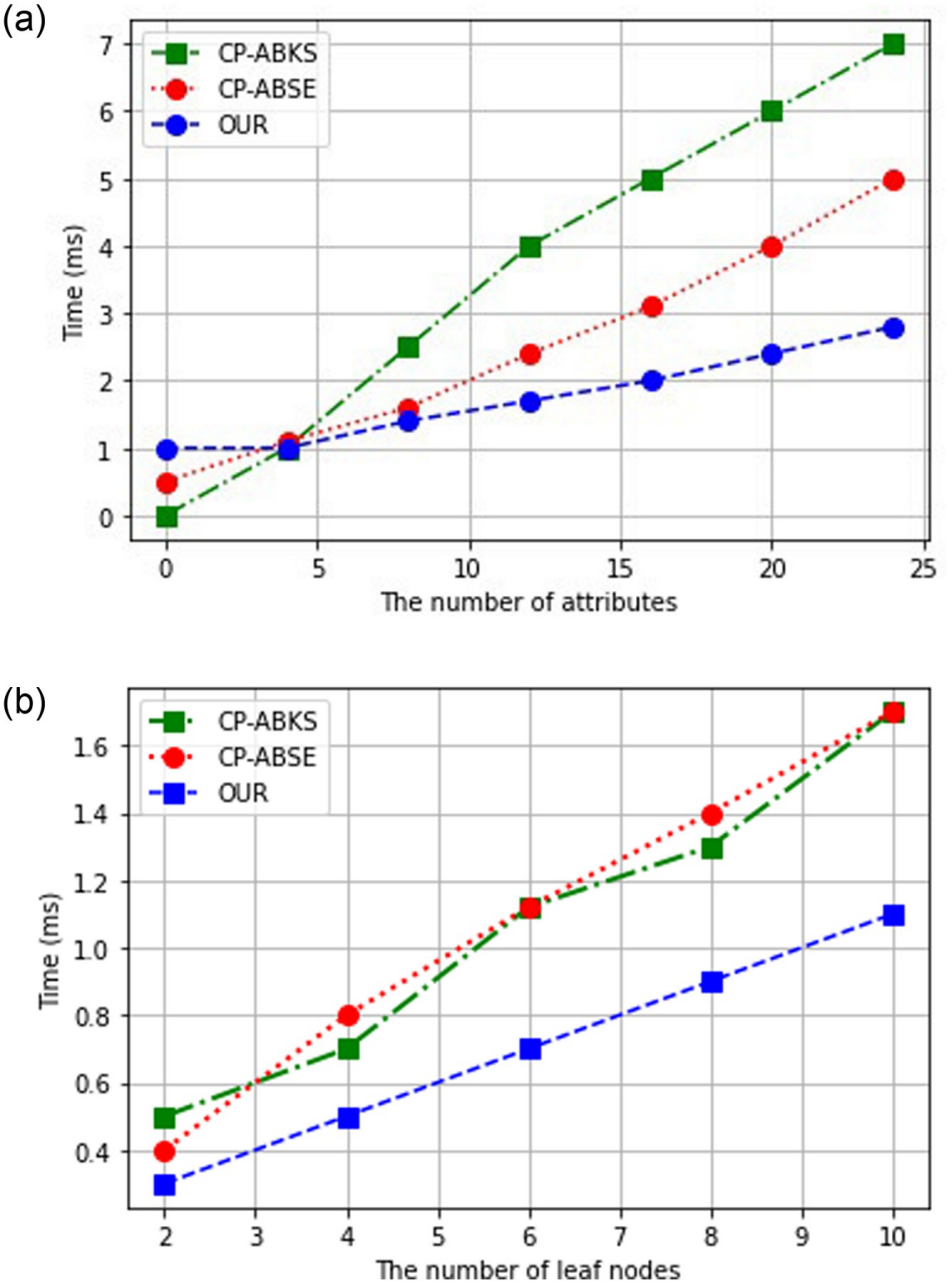
The storage cost. (a) KeyGen algorithm, (b) EncInd algorithm.

KeyGen algorithm is run by trusted attribute authority to label each claimed attribute of the data user to its secret key components, then through a secure channel transfer to its indented data users. As demonstrated in Fig 6a, the computation cost of all schemes linearly increased with the increase in the number of attributes. Compared to CP-ABSE and CP-ABKS, we can observe that our proposed scheme requires less computation time as we increase the number of attributes in the data user list. Its better performance is due to the lesser exponentiation operation for keys generation than the other two schemes.

### Evaluation of EncInd algorithm

This algorithm is run by the data owner and output a secure keyword index accessible under access control policy sets by its data owner. This acts as a specific clue for a cloud server to relate any search query keyword from data users without revealing any underlying encrypted keyword. More specifically, the cloud server performs the search operation against the encrypted keyword to find out the relevant encrypted document. Fig 6b shows that the computation time for each algorithm linearly increases as we increase the number of attributes attached to the leaf nodes in the access control policy. We can also see from Fig 6b that our proposed scheme outperforms the two schemes because of its lesser computation burden on data users.

### Evaluation of TokenGen algorithm

**Fig 7:**
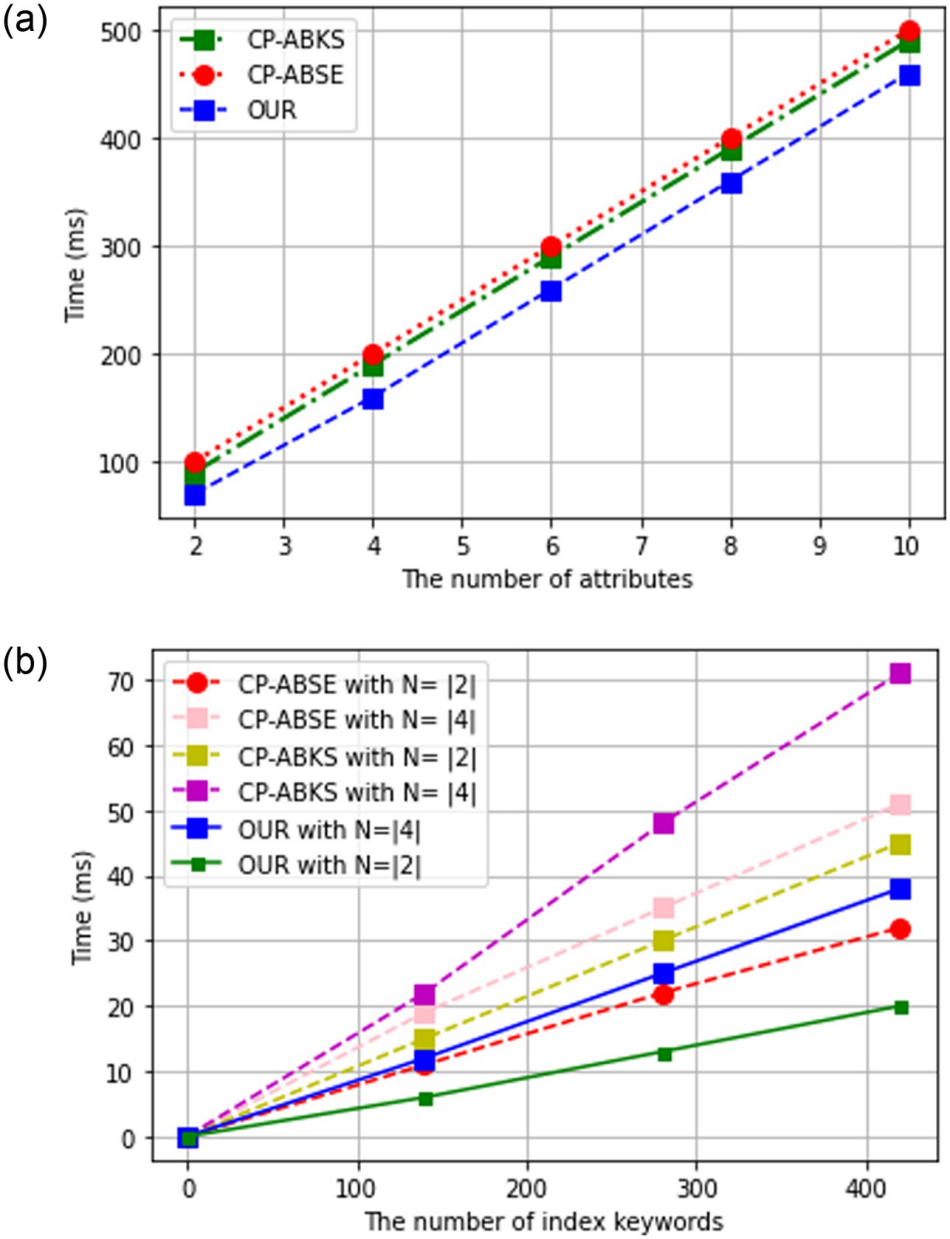
The storage cost. (a) TokenGen algorithm, (b) Search algorithm.

The data user runs this algorithm to encrypt his keyword in a trapdoor for secure searching on the cloud. Fig 7a shows the time taken by each scheme for the encryption of the query keyword. Both CP-ABSE and CP-ABKS are linearly proportional to the data user’s attributes set, which incur high computation overhead. Our proposed scheme embeds constant delegated key components instead of each individual’s attribute. In this way, our proposed scheme avoids the linearity problem of ABE and performs efficiently.

### Evaluation of search algorithm

When the cloud server receives the trapdoor query from the data user, it needs to perform two kinds of checks; first, it needs to find out if this data user possesses the attributes corresponding to each leaf node of T. Second, check out if it has the stored index ${ℐ}_{w}$ equal to the query token $T_{q},w=q$. Fig 7b shows the average running time for both these steps. We run each scheme for a different value of *N*, where *N* is the set of attributes that is labeled with the access tree T of the ciphertext. From Fig 7b, we can see that the running time for all the schemes linearly increases for both the index keywords and *N*. With only three operations in the token confirmation phase and complete avoidance of costly pairing operation in access conformation, our proposed scheme performs better in searching, which is the key performance indicator for any searching schemes.

## Conclusion

This paper proposed an EFG-KSS scheme, free from costly bilinear pairing operations during the search and expensive Lagrange interpolation for secret reconstruction. Our scheme also supports the updation of the access control policy without completely re-encrypt the ciphertext. The security proof is provided under the Decisional Bilinear Diffe-Helmen (DBDH) assumption. The experimental results show that the proposed scheme gains better communication overhead along with low computation costs. As future work, we would like to make it privacy-preserving ABKS, enabling the data owner to encrypt the data without explicitly embedding the access control structure in the ciphertext.
